# Harnessing the human gut microbiota: an emerging frontier in combatting multidrug-resistant bacteria

**DOI:** 10.3389/fimmu.2025.1563450

**Published:** 2025-03-17

**Authors:** Wenwen Ding, Yiwen Cheng, Xia Liu, Zhangcheng Zhu, Lingbin Wu, Jie Gao, Wenhui Lei, Yating Li, Xin Zhou, Jian Wu, Yongtao Gao, Zongxin Ling, Ruilai Jiang

**Affiliations:** ^1^ Department of Anesthesiology, Affiliated Hospital of Nantong University, Nantong, Jiangsu, China; ^2^ Medical School of Nantong University, Nantong, Jiangsu, China; ^3^ Collaborative Innovation Center for Diagnosis and Treatment of Infectious Diseases, State Key Laboratory for Diagnosis and Treatment of Infectious Diseases, National Clinical Research Center for Infectious Diseases, The First Affiliated Hospital, School of Medicine, Zhejiang University, Hangzhou, Zhejiang, China; ^4^ Department of Intensive Care Unit, The First Affiliated Hospital, School of Medicine, Zhejiang University, Hangzhou, Zhejiang, China; ^5^ Department of Preventive Medicine, School of Public Health and Management, Wenzhou Medical University, Wenzhou, Zhejiang, China; ^6^ Department of Intensive Care Unit, Lishui Second People’s Hospital, Lishui, Zhejiang, China; ^7^ Jinan Microecological Biomedicine Shandong Laboratory, Jinan, Shandong, China; ^8^ Department of Genetics, Stanford University School of Medicine, Stanford, CA, United States; ^9^ Stanford Center for Genomics and Personalized Medicine, Stanford, CA, United States; ^10^ Stanford Diabetes Research Center, Stanford, CA, United States; ^11^ The Jackson Laboratory for Genomic Medicine, Farmington, CT, United States; ^12^ Department of Clinical Laboratory, Suzhou Municipal Hospital, Suzhou, Jiangsu, China

**Keywords:** gut microbiota, antimicrobial resistance, colonization resistance, bacteriophage, probiotics

## Abstract

Antimicrobial resistance (AMR) has become a major and escalating global health threat, undermining the effectiveness of current antibiotic and antimicrobial therapies. The rise of multidrug-resistant bacteria has led to increasingly difficult-to-treat infections, resulting in higher morbidity, mortality, and healthcare costs. Tackling this crisis requires the development of novel antimicrobial agents, optimization of current therapeutic strategies, and global initiatives in infection surveillance and control. Recent studies highlight the crucial role of the human gut microbiota in defending against AMR pathogens. A balanced microbiota protects the body through mechanisms such as colonization resistance, positioning it as a key ally in the fight against AMR. In contrast, gut dysbiosis disrupts this defense, thereby facilitating the persistence, colonization, and dissemination of resistant pathogens. This review will explore how gut microbiota influence drug-resistant bacterial infections, its involvement in various types of AMR-related infections, and the potential for novel microbiota-targeted therapies, such as fecal microbiota transplantation, prebiotics, probiotics, phage therapy. Elucidating the interactions between gut microbiota and AMR pathogens will provide critical insights for developing novel therapeutic strategies to prevent and treat AMR infections. While previous reviews have focused on the general impact of the microbiota on human health, this review will specifically look at the latest research on the interactions between the gut microbiota and the evolution and spread of AMR, highlighting potential therapeutic strategies.

## 1 Introduction

Antimicrobial resistance (AMR) is a global health crisis, largely driven by the overuse and misuse of antibiotics. This has led to a significant rise in drug-resistant infections, contributing to millions of deaths each year (1, 2). Alarmingly, projections suggest that by 2050, AMR could be responsible for 10 million deaths annually (3, 4). The situation is made worse by the slow pace at which new antibiotics are developed and the growing presence of ESKAPE pathogens—*Enterococcus faecium*, *Staphylococcus aureus*, *Klebsiella pneumoniae*, *Acinetobacter baumannii*, *Pseudomonas aeruginosa*, and *Enterobacter* species—which are leading contributors to AMR-related mortality (2, 5). These pathogens, although typically harmless and existing in a symbiotic relationship with the host, can become opportunistic in the event of immune system disruptions. This leads to infections that result from disturbances in the gut microbiota. Alterations in microbial composition, changes in bacterial metabolic activity, and shifts in local bacterial distribution further exacerbate gut dysbiosis, increasing the risk of infections. Furthermore, the slow pace of new antimicrobial drug development has failed to keep up with the rapid rise in AMR. This growing gap between rising resistance and limited new antibiotics highlights the urgent need for innovative strategies to manage infections and combat AMR.

As a response to this urgent challenge, researchers are increasingly turning to the human gut microbiota as a promising ally in the fight against AMR. The gut microbiota plays a vital role in supporting the immune system and protecting the body from infections (6–8). It helps regulate immune responses and maintains a delicate balance of microorganisms. However, disruptions to this balance—often caused by antibiotics or illness—can allow harmful, antibiotic-resistant pathogens to overgrow (5). Recent studies highlight how microbiota-based therapies, such as fecal microbiota transplantation (FMT) and probiotics, can help restore a healthy microbial balance and fight off drug-resistant infections. For example, Kellogg et al. find that succinate-producing microbiota drive tuft cell hyperplasia to protect against *Clostridioides difficile* (9). FMT has shown promise in treating recurrent *C. difficile* infections by outcompeting harmful bacteria and re-establishing a balanced microbiota, with approval from the U.S. FDA (10). Beyond FMT, other microbiota-based strategies, including prebiotics, synbiotics, postbiotics, and bacteriophage, are also being explored as potential weapons in the fight against AMR. These therapies work in various ways, such as fostering the growth of beneficial microbes, enhancing immune function, and directly inhibiting the growth of harmful bacteria.

This review will delve into the molecular mechanisms through which the gut microbiota influences AMR. It will examine the role of metabolites produced by gut bacteria, immune modulation, and competitive inhibition in shaping the body’s response to infections. Moreover, we will explore the diversity of the gut microbiota in different AMR bacterial infections and assess the potential of microbiota-targeting therapies as a promising approach to combatting AMR.

## 2 Mechanisms of gut microbiota in combating MDR infections

**Figure 1:**
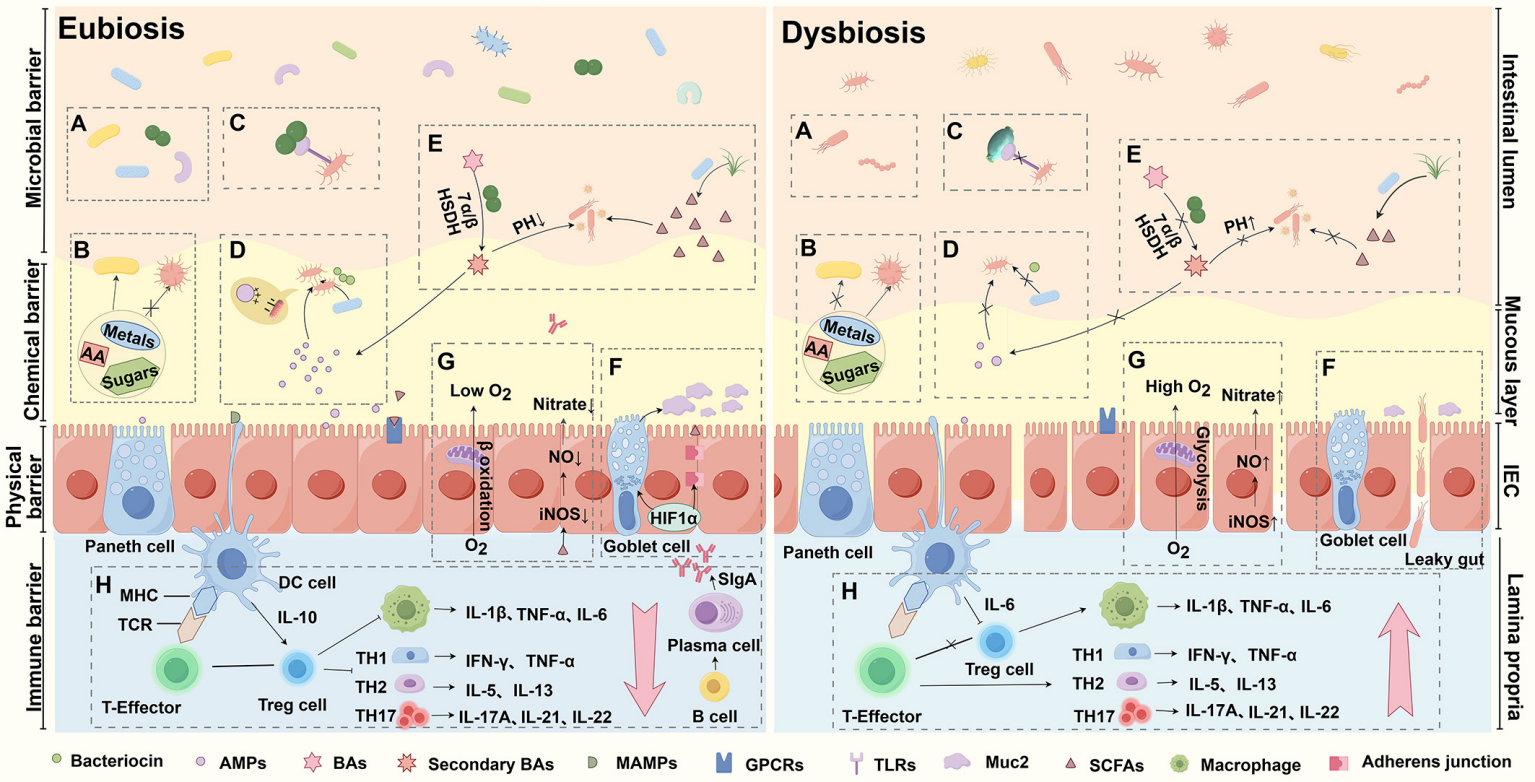
Mechanisms of gut microbiota in combating MDR infections. **(A)** Niche Exclusion: Dysbiosis reduces microbiota diversity, weakening pathogen exclusion. **(B)** Nutritional Competition: Healthy microbiota outcompetes pathogens for nutrients, thereby limiting the growth of pathogens by outcompeting them for resources. **(C)** Contact-Dependent Inhibition (CDI): Bacteria deliver toxins via cell contact, inhibiting or killing neighboring cells. **(D)** Antimicrobial Peptides (AMPs): Short-chain fatty acids (SCFAs) induce AMP synthesis, regulating microbiota and exerting antimicrobial effects. **(E)** Inhibitory Metabolites: SCFAs and secondary bile acids (BAs) inhibit pathogens or modulate immunity. **(F)** Mucosal Barrier: Dysbiosis reduces Muc2 production, compromising the intestinal barrier and increasing infection risk. **(G)** Oxygen Limitation: Inflammation, driven by iNOS and NADPH oxidase, limits oxygen availability during infection. **(H)** Immune Modulation: Microbiota activates Toll-like receptors (TLRs), crucial for immune signaling and defense.

The human microbiota has established a mutualistic symbiosis with its host, contributing to overall health through metabolic support, immune modulation, and protection against pathogens (11, 12). A key mechanism by which the gut microbiota combats multidrug-resistant (MDR) infections is colonization resistance (CR), which prevents the colonization and overgrowth of both external pathogens and resident pathobionts (13). The gut microbiota employs a multifaceted defense strategy to protect the host, including nutritional competition, niche exclusion, contact-dependent inhibition, and the production of antimicrobial peptides and inhibitory metabolites (**Figure 1**). Furthermore, the microbiota contributes to mucosal barrier integrity, creates oxygen-limited environments that are inhospitable to many pathogens, and modulates immune responses to enhance immune tolerance and protection (13–15). Collectively, these mechanisms form a robust defense system that restricts the establishment and proliferation of harmful microorganisms, thereby safeguarding gut health and preventing MDR infections. A deeper understanding of how the gut microbiota limits pathogen colonization can inform the development of innovative therapeutic strategies to address the growing challenge of AMR. Harnessing the potential of the gut microbiota to enhance CR offers a promising avenue to combat MDR infections and mitigate the global threat of AMR.

### 2.1 Nutritional competition

The gut microbiota plays a critical role in preventing drug-resistant bacterial infections through nutritional competition, a mechanism that revolves around the depletion of essential resources required for bacterial growth. By efficiently consuming nutrients such as carbon sources, nitrogen sources, and metal ions, the gut microbiota limits their availability to potential pathogens, thereby restricting their growth and colonization. A diverse microbial community enhances this competitive edge, as highlighted by Spragge et al., who demonstrated that a rich microbial ecosystem intensifies nutrient competition, effectively outcompeting harmful pathogens (16). For example, the commensal bacterium *Klebsiella michiganensis* has been shown to outcompete *Escherichia coli in vitro* by depleting essential nutrients, limiting *E. coli*’s survival and growth (17). Similarly, commensal strains of *E. coli*, such as HS and Nissle 1917, inhibit the colonization of pathogenic *E. coli* O157:H7 by consuming sugars critical for the pathogen’s survival (18, 19). Genetic studies have further illuminated the role of nutrient competition in CR. For example, the gut microbiota’s uptake of dietary amino acids, such as tryptophan and arginine, plays a crucial role in preventing infections by pathogens like *Citrobacter rodentium*. However, a high-protein diet can enhance *C. rodentium* colonization in mice, underscoring the influence of nutrient availability on microbial dynamics (20). This balance is also evident in the case of *C. difficile*, where the gut microbiota competes for essential amino acids, restricting the pathogen’s proliferation (21). Interestingly, *C. difficile* attempts to counteract this by upregulating indole, a tryptophan metabolite, to promote its growth. Nevertheless, the microbiota’s efficient nutrient utilization limits the pathogen’s ability to exploit this strategy, further preventing its overgrowth (22). In addition to organic nutrients, metal ions such as iron, zinc, and manganese are critical for microbial survival and virulence. These metals are often scarce in the gut and are further sequestered by the host during inflammatory responses to limit pathogen access. Commensal bacteria, such as *E. coli* strain Nissle 1917, employ specialized mechanisms to acquire iron, providing protection against *Salmonella* infections (23). Conversely, pathogens like *Vibrio cholerae* and *Campylobacter jejuni* rely on efficient zinc uptake to survive and thrive in the gastrointestinal tract (24). By outcompeting pathogens for these vital nutrients, the gut microbiota establishes a robust defense mechanism, highlighting the critical role of nutritional competition in preventing infections and maintaining gut health. This understanding offers valuable insights into developing strategies to enhance CR and combat the growing threat of multidrug-resistant infections.

### 2.2 Niche exclusion

Niche exclusion is a critical strategy employed by the gut microbiota to limit pathogen colonization by occupying physical niches, depleting essential nutrients, or producing inhibitory metabolites. These competitive interactions are essential for maintaining gut homeostasis and preventing infections. Microbial species, particularly those genetically similar, often engage in direct competition. For instance, *K. pneumoniae*, *Citrobacter*, and *Clostridium* species inhibit the colonization of more pathogenic relatives through competitive exclusion (25–27). The “nutrient ecotope” hypothesis, introduced by Freter et al., provides a framework for understanding microbial competition in the gut. It posits that the availability of specific nutrients shapes the abundance and distribution of microbial species, with gut microbes actively consuming resources to define their niche (28). For example, Munehiro et al. isolated a symbiotic bacterial complex from healthy human fecal samples that inhibited Enterobacteriaceae, particularly *Klebsiella*, by regulating the availability of gluconates, a key nutrient for bacterial growth (19). Beyond nutrient competition, microbial species can coexist by acquiring novel traits. Sweeney et al. demonstrated that two closely related *E. coli* strains coexisted in the same niche by acquiring a high-affinity gluconate transporter, enabling them to outcompete other bacteria for the same carbon source (29). This highlights how adaptive resource uptake can shift competitive dynamics. Adhesion to mucosal surfaces is another crucial aspect of niche exclusion, as it prevents the colonization of exogenous pathogens. Kasper et al. showed that the IgA response facilitates *Bacteroides fragilis* in occupying a stable mucosal niche, where it inhibits harmful bacteria (30). This underscores the interplay between microbial communities and host immune responses in mediating niche occupancy. Additionally, some microbial species modify their environment to enhance colonization while excluding competitors (30, 31). For example, symbiotic bacteria limit *Salmonella typhimurium* colonization by occupying mucosal space and depleting nutrients like carbon sources and oxygen (32). Collectively, niche exclusion mechanisms—through resource competition, adhesion site occupation, and environmental modification—form a robust defense system that maintains microbial homeostasis and protects the host from infections. These insights highlight the intricate competitive dynamics within the gut microbiota and their role in preventing pathogen colonization.

### 2.3 Contact-dependent inhibition

Contact-dependent inhibition (CDI) is a sophisticated bacterial defense mechanism that enables bacteria to outcompete rivals through direct cell-to-cell interactions. This system involves the delivery of toxic effectors into neighboring cells, leading to growth inhibition or cell death. Initially discovered in *E. coli* strain EC93 in 2005, the CDI locus was found to contain three genes—*CdiB*, *CdiA*, and *CdiI*—which are sufficient to confer CDI activity to *E. coli* K-12 (33). Since then, CDI systems have been identified in various bacteria, including *Enterobacteriaceae*, *Moraxellaceae*, *Pasteurellaceae*, *Neisseria meningitidis*, *Yersinia pestis*, *Dickeya dadantii*, *Enterobacter cloacae* and *Photorhabdus luminescens* (34–37). The CDI system is centered on the CdiA protein, which acts as the effector by disrupting cellular processes, such as DNA degradation, in target cells (38). CdiB facilitates the transport of CdiA across the bacterial membrane, while CdiI provides immunity to the CDI^+^ bacterium by protecting it from its own toxins (39). Upon contact, the CdiB/CdiA two-partner secretion system delivers toxins directly into neighboring cells, inhibiting their growth or causing cell death (40). This mechanism allows bacteria to outcompete rivals in shared environments, such as during infection or biofilm formation (41). Consequently, the CDI system is increasingly viewed as a bacterial “weapon” system used to dominate microbial ecosystems (42, 43).

Another critical antibacterial mechanism is the type VI secretion system (T6SS), employed by many Gram-negative pathogens to inject toxic proteins into neighboring cells (44–47). 6SS-mediated interactions play a vital role in interbacterial competition, particularly among *Bacteroides* species in the gastrointestinal tract, where they compete for space and resources (48, 49). For example, *B. fragilis* inhibits *B. polymorphicus* growth *in vitro* through a T6SS-dependent mechanism (45) and exhibits competitive resistance against pathogenic strains (50, 51). These findings highlight the T6SS’s role in maintaining gut homeostasis and protecting against pathogenic invasion. Other secretion systems, such as Type IV and Type VII, also mediate antibacterial interactions through protein translocation and cell killing (52, 53), highlighting the importance of antagonistic interactions in the gut. Collectively, these systems demonstrate how bacteria have evolved complex strategies to shape microbial ecosystems and promote their survival. The CDI and T6SS systems represent potential targets for novel antimicrobial therapies aimed at disrupting toxin-delivery pathways, offering promising avenues to combat MDR infections and restore microbial balance in the gut (**Table 1**).

**Table 1:** CDI protein and its mechanism of action.

Strains	CDI	Action mechanism	References
Firmicutes	LXG polymorphic toxin	•LXG toxins in *Streptococcus intermedius* inhibit Firmicutes via cell contact and Esx secretion	(52)
*Group B Streptococcus*	T7SSs	•GBS secretes LXG toxins via T7SS, killing or inhibiting neighboring bacteria by disrupting cell walls or metabolic pathways, gaining a competitive edge in microbial communities •GBS encodes an immune protein that pairs with LXG toxins, protecting itself from self-harm	(53)
*E. coli*	CdiA-CT	•N-terminal “entry” domains hijack membrane proteins to facilitate toxin assembly into the lipid bilayer. •CDI ionophores in *E. coli* isolates are grouped into six major classes based on entry domain structures •Ionophore domains show significant intra-group sequence variation, especially at CdiI interaction sites	(39)
*E. coli* EC93	CdiA	•BamA-CdiA interaction relies on a few non-conserved amino acids on BamA’s extracellular surface, extending near its lateral gate •BamA’s lateral gate can open without disrupting CdiA interaction	(38)
*Acinetobacter baumannii*, *Escherichia coli*	CdiB/CdiA	•CdiB transporters adopt a TpsB fold, with a 16-stranded β-barrel occluded by an N-terminal α-helix and extracellular loop 6, which exhibit distinct conformations •H1 and the DxxG motif are present in CdiB/TpsB transporters but absent in BamA/TamA proteins	(40)

CDI, Contact-dependent inhibition; LXG,Leu-X-Gly; T7SSs,Type VII secretion system; Tps, Type Vb secretion system.

### 2.4 Antimicrobial peptides

Antimicrobial peptides (AMPs) are essential components of the innate immune system, produced by host epithelial cells, neutrophils, and other immune cells such as mast cells and Paneth cells (54, 55). These peptides exhibit bactericidal, anti-inflammatory, and anti-endotoxin properties, serving as a first line of defense against microbial threats (56). AMPs disrupt microbial membranes and inhibit pathogen growth through diverse mechanisms. For example, nisin, produced by *Lactococcus lactis*, targets lipid II (a cell wall precursor) in pathogenic bacteria, forming pores in their membranes, causing cell content leakage, and disrupting membrane integrity (57). Similarly, RegIIIβ and RegIIIγ contribute to gut defense by interacting with the G protein-coupled receptor GPR43 (58, 59). AMPs also inhibit bacterial growth through specific molecular interactions. Microcin J25 targets *E. coli* RNA polymerase, suppressing bacterial replication (60), while colicins, produced by *E. coli*, degrade tRNA or disrupt proton gradients to inhibit pathogenic metabolism (61). Beyond their direct antimicrobial effects, AMPs regulate the gut microbiota, preventing the overgrowth of opportunistic pathogens and supporting immune homeostasis (62). For instance, oral administration of AMPs has been shown to alleviate intestinal inflammation induced by Enterohemorrhagic *E. coli* and modulate the gut microbiota (63). Bacteriocins, a subclass of AMPs produced by bacteria, selectively target closely related species to inhibit their growth or induce cell death, promoting competitive exclusion (64–66). For example, nisin is effective against Gram-positive bacteria like *S. aureus* and *Listeria monocytogenes* (67, 68), while class II bacteriocins such as Actifencin and Bacteroidetocin A selectively inhibit *Lactobacillus* and *Bacteroides* species, respectively, preserving microbiota diversity (69). Bacteriocins also enhance the resistance of beneficial bacteria, such as *E. coli* Nissle, against harmful microbes (70, 71). This specificity within microbial communities helps maintain balance and offers therapeutic potential, such as protecting against *L. monocytogenes* infections via strains like *L. salivarius* UCC 118 (72, 73). Therefore, AMPs play a dual role in defending against infections and shaping the gut microbial landscape. Their ability to modulate microbial abundance and diversity is critical in preventing pathogen overgrowth and maintaining gut homeostasis (74). These properties highlight the potential of AMPs as therapeutic agents to combat infections and restore microbial balance.

### 2.5 Inhibitory metabolites

The gut microbiota produces a variety of inhibitory metabolites, including short-chain fatty acids (SCFAs) and secondary bile acids (BAs), which play critical roles in directly inhibiting pathogen growth and modulating host immune responses to maintain gut health (75, 76). SCFAs, primarily derived from the fermentation of dietary fibers by the gut microbiota (75), exhibit direct antimicrobial effects by disrupting bacterial membrane integrity and intracellular pH homeostasis. This mechanism inhibits pathogens such as *E. coli* and *Salmonella* under acidic conditions (77). Beyond their antimicrobial activity, SCFAs exert immunomodulatory effects, including promoting the differentiation of regulatory T cells (Tregs), which help mitigate excessive inflammation (78). For example, butyrate increases Foxp3^+^ Tregs in the spleen and lymph nodes of antibiotic-treated mice (79), while propionate suppresses IL-17 production in intestinal γδT cells (80). SCFAs also enhance the intestinal epithelial barrier by promoting the integrity and function of epithelial cells, thereby strengthening mucosal immunity and preventing pathogen adhesion (81, 82). Additionally, SCFAs provide energy to intestinal epithelial cells, supporting normal cellular respiration and preventing the establishment of pathogens like *Citrobacter* (83). A key mechanism by which SCFAs maintain gut homeostasis is through the regulation of hypoxia-inducible factor 1-alpha (HIF-1α). Butyrate stabilizes HIF-1α by inhibiting histone deacetylases (HDACs) and activating G-protein-coupled receptors such as GPR43 and GPR109A. This process upregulates tight junction proteins like occludin and claudin-1, enhancing intestinal barrier function and reducing permeability (84). HIF-1α activation also promotes the secretion of β-defensins by intestinal epithelial cells, inhibiting the overgrowth of *E. coli* and *Salmonella* while supporting beneficial bacteria like *Lactobacillus* and *Bifidobacterium* (85).

Secondary BAs, such as deoxycholic acid (DCA) and lithocholic acid (LCA), are produced by specific gut microbiota species through the metabolism of primary BAs. These metabolites exhibit antimicrobial properties, inhibiting pathogens like *C. difficile* (86, 87) and *Salmonella* while selectively promoting beneficial bacteria (88, 89). DCA exerts bactericidal effects by lowering intracellular pH and disrupting bacterial membrane integrity (90, 91), while LCA indirectly enhances gut defense by stimulating the transcription of antimicrobial peptides like LL-37 (92). Secondary BAs also modulate immune responses, with certain *Bacteroidetes* species promoting Treg differentiation through BA modification, further supporting immune homeostasis (93, 94). Disruptions to the gut microbiota, such as those caused by antibiotics or dietary changes, can impair the production of these protective metabolites, increasing susceptibility to infections and pathogen overgrowth (95). Therefore, maintaining a balanced gut microbiota is essential for ensuring the continuous production of inhibitory metabolites that limit pathogen growth and support immune homeostasis. These insights highlight the therapeutic potential of modulating SCFAs and secondary BAs to combat infections and promote gut health.

### 2.6 Mucosal barrier

The gut mucosal barrier is a complex, multi-layered defense system that integrates physical, microbiological, immune, and chemical components to protect the gastrointestinal tract from pathogens. The intestinal epithelial barrier, including its mucus layer, serves as a physical shield against harmful microorganisms (96). Pathogens like *E. coli* can attach to epithelial cells to initiate infections, but commensal microorganisms strengthen the mucosal barrier to prevent this. Mucin 2 (Muc2), a key component of the mucus layer, is central to maintaining gut barrier integrity. Germ-free mice, which lack a normal gut microbiota, produce less Muc2 and are more susceptible to infections (97, 98). HIF-1α, a transcription factor responsive to low oxygen conditions, plays a critical role in regulating Muc2 secretion by goblet cells. This forms a protective mucus layer that prevents direct pathogen contact with epithelial cells (99). *E. coli* can also stimulate Muc2 production, highlighting the microbiota’s role in host defense. Muc2-deficient mice are more vulnerable to pathogen colonization and experience severe infections, such as those caused by *L. monocytogenes* (100). Diet significantly influences mucosal barrier integrity. Mice fed a fiber-free diet develop a thinner mucus layer, increasing susceptibility to pathogens like *L. citrobacter* (101, 102). Additionally, L-fucose, a sugar produced by gut bacteria, reduces the virulence of pathogens like *Burkholderia citriodora*, further demonstrating the microbiota’s role in modulating immune responses and pathogen defense (103). HIF-1α also regulates tight junctions between intestinal epithelial cells, enhancing barrier function. It upregulates tight junction proteins such as occludin, claudin-1, and ZO-1, preventing the penetration of pathogens and toxins (84). Disruption of these junctions by pathogens like *C. difficile*, which produces toxins TcdA and TcdB, increases intestinal permeability and facilitates pathogen invasion (104, 105). This underscores the critical role of the gut microbiota in maintaining epithelial barrier function. The gut microbiota further supports the mucosal barrier by producing metabolites that enhance immune function and inhibit pathogenic microbes (76). HIF-1α promotes energy metabolism in intestinal epithelial cells under hypoxic conditions, upregulating genes like GLUT1 and LDHA to reinforce barrier integrity (84). In summary, the mucosal barrier relies on a combination of mechanisms, including Muc2 secretion, tight junction regulation, and energy metabolism, to maintain gut homeostasis and protect against infections. These processes highlight the intricate interplay between the gut microbiota and host defenses in preserving intestinal health.

### 2.7 Oxygen-limited conditions

The gut is characterized by oxygen-limited conditions, with oxygen levels sharply decreasing in the deeper layers of the mucosal surface. This anaerobic environment is critical in shaping the composition and function of the gut microbiota. Pathogens that thrive in higher oxygen environments, such as *E. coli*, *Salmonella*, and *C. difficile*, struggle to establish themselves in this setting, while commensal bacteria, well-adapted to low oxygen, outcompete pathogens for space and nutrients (13, 106). Oxygen-limited conditions promote the growth of microbial species that produce SCFAs, which support mucosal immunity and strengthen the gut barrier. However, during infections, the oxygen balance can shift, creating a more favorable environment for pathogens. Infections trigger an inflammatory response, primarily through the activation of inducible nitric oxide synthase (iNOS) and NADPH oxidase. iNOS, encoded by the *Nos2* gene, produces nitric oxide by oxidizing L-arginine, while NADPH oxidase generates reactive oxygen species (ROS), contributing to inflammation (15, 107). Pathogens such as *S. typhimurium* and *E. coli* can induce inflammation and dysbiosis, increasing oxygen and nitrate availability in the intestinal lumen. These molecules serve as terminal electron acceptors for pathogenic bacteria, promoting their survival and growth (108, 109). Commensal bacteria play a vital role in maintaining the gut’s hypoxic state, which limits pathogen expansion. For example, commensals that produce butyrate via β-oxidation reduce oxygen levels by stimulating aerobic respiration in intestinal cells (13), creating an oxygen-poor environment that hinders pathogen growth. However, in conditions of dysbiosis, where butyrate production is diminished, oxygen levels rise, supporting the growth of pathogens like *S. typhimurium* (110, 111). Inflammation and dysbiosis can further reduce butyrate-producing bacteria, exacerbating oxygen levels and promoting the growth of pathogenic anaerobes (112, 113). Therefore, oxygen-limited conditions are essential for maintaining a healthy gut microbiota and providing CR against pathogens.

### 2.8 Immune modulation

The gut is a central hub of the immune system, housing approximately 70–80% of the body’s immune cells (114). It acts as a critical interface between the body and external pathogens, with the gut microbiota playing a pivotal role in maintaining immune homeostasis. This intricate interplay between the microbiota and the mucosal immune system is essential for both immune tolerance and defense mechanisms. The gut microbiota influences the development, maturation, and function of the immune system, impacting both innate and adaptive immunity (114). It educates the immune system to distinguish between harmful pathogens and harmless substances, such as food and beneficial microbes. A balanced microbiota promotes a tolerogenic immune response, preventing excessive activation that could lead to chronic inflammation or autoimmune diseases. One key mechanism by which the gut microbiota interacts with the immune system is through the activation of pattern recognition receptors (PRRs), such as Toll-like receptors (TLRs) (115). These receptors trigger the release of antimicrobial peptides, cytokines, and chemokines, initiating inflammatory responses to combat infections (116, 117). Additionally, the microbiota stimulates the production of IgA, an antibody that neutralizes pathogens, controls harmful bacterial colonization, and supports beneficial microbes (118). The absence of secretory IgT, an ancient immunoglobulin involved in mucosal immunity, disrupts the microbiota in species like rainbow trout, making them more susceptible to infections (119). This highlights the evolutionary importance of immune responses in maintaining gut microbiota balance. The gut microbiota also plays a crucial role in the differentiation of Tregs, which suppress excessive immune reactions and prevent inflammation (120). Conversely, it can stimulate the production of Th17 cells, which defend against extracellular pathogens but, when overactivated, contribute to inflammatory conditions. A balanced microbiota maintains the equilibrium between Tregs (anti-inflammatory) and Th17 cells (pro-inflammatory), supporting gut homeostasis. Beneficial microbes produce SCFAs, such as butyrate, propionate, and acetate, which possess anti-inflammatory properties and enhance Treg function (121, 122). SCFAs also contribute to immune regulation by promoting the survival of memory T cells and maintaining gut barrier integrity, preventing “leaky gut” and systemic infections (78). Overall, the gut microbiota is essential for immune homeostasis. Dysbiosis can increase susceptibility to pathogen colonization, infections, and inflammatory or autoimmune disorders.

## 3 Roles of gut microbiota in different types of MDR infections

**Figure 2:**
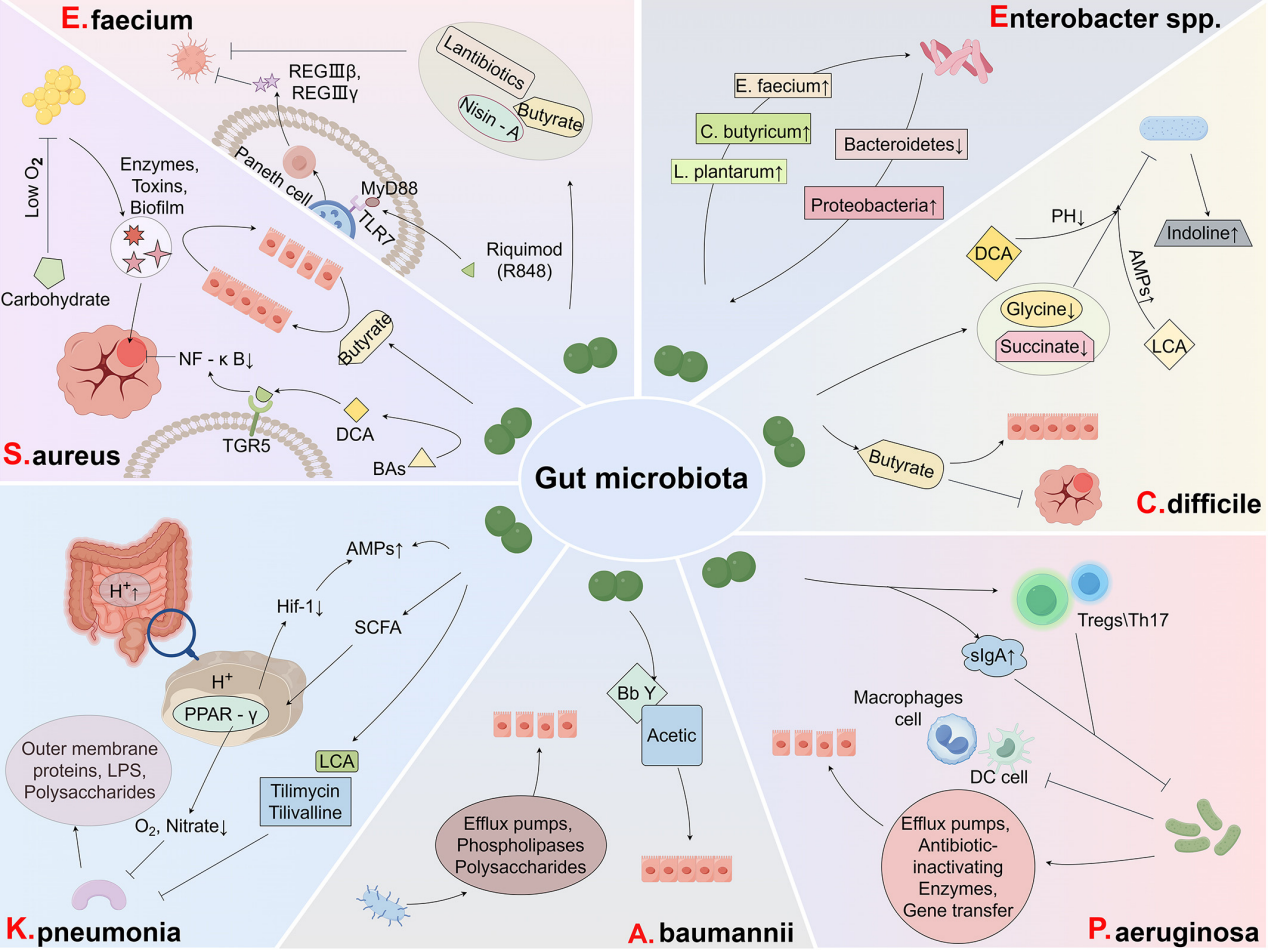
Roles of gut microbiota in different types of MDR infections. *Enterococcus faecium*: The gut microbiota resists *E. faecium* by secreting antibacterial substances that inhibit its growth. *Staphylococcus aureus*: The gut microbiota counters *S. aureus* by restoring the mucosal barrier and suppressing inflammation, thereby limiting bacterial colonization. *Klebsiella pneumoniae*: The release of beneficial metabolites and induction of intracellular acidification by the gut microbiota inhibit the growth of *K. pneumoniae*, curbing its spread. *Acinetobacter baumannii*: Restoration of the mucosal barrier by the gut microbiota prevents the invasion and colonization of *A. baumannii*, enhancing host defense. *Pseudomonas aeruginosa*: Through immune modulation, the gut microbiota strengthens the host’s immune response, effectively suppressing *P. aeruginosa* infections. *Clostridium difficile*: The gut microbiota mitigates *C. difficile* infections by reducing inflammation, regulating microbial metabolites, and restoring mucosal barrier integrity. *Enterobacter* spp.: A diverse gut microbiota inhibits *Enterobacter* species, contributing to the prevention of infections and maintaining microbial balance.

The escalating global threat of AMR, fueled by the widespread misuse and overuse of antimicrobials, has underscored the urgent need for innovative solutions. Multidrug-resistant (MDR) pathogens, including *C. difficile* and the ESKAPE (ESKAPCE) group, pose significant challenges to modern healthcare systems (123). These pathogens exhibit resistance to conventional antibiotics, leading to treatment failures, recurrent infections, prolonged hospitalizations, and increased mortality rates. The scarcity of effective therapeutic options exacerbates this crisis (124). Emerging evidence highlights the pivotal role of the gut microbiota in modulating the spread and progression of AMR (124). The gut microbiota, a complex microbial ecosystem, can either facilitate or impede the colonization of drug-resistant pathogens. Understanding the intricate interactions between the gut microbiota and MDR bacteria is critical for developing novel strategies to combat resistant infections (**Figure 2**, **Table 2**).

**Table 2:** Roles of gut microbiota in different types of MDR infections.

Drug-resistant bacteria	Gut microbiota/metabolites	Action mechanism	Major findings	References
*E. faecium*	*E.coli* Nissle 1917	Antimicrobial peptides	•*E.coli* Nissle 1917 expression Bacteroidetocin A (#22) effectively inhibited *Enterococcus faecalis* ATCC 19433.	(69)
	*Clostridium bolteae*, *Blautia producta* (BP_SCSK_), *Bacteroides sartorii* and *Parabacteroides distasonis*	Antimicrobial peptides	•↑BPS_CSK_ lantibiotic •Inhibited VRE *in vitro* •Without disturb the gut microbiota	(125)
	Two *Lactobacillus* strains (Y74 and HT121)	Niche exclusion, antimicrobial peptides	•Defense-related genes (defensin α, Apoa1, and RegIII) •Restore the diversity of gut microbiota	(126)
	*Bacteroides*	Inhibitory metabolites	•↑butyric acid •gut microbiota changes	(127)
*S. aureus*	*Staphylococcus lugdunensis*	Nutritional competition	•*Staphylococcus ludunensis* exploits *S. aureus*-secreted iron carriers, depriving *S. aureus* of iron and inhibiting its growth	(128)
	*NaB*	Inhibitory metabolites	•↓inflammatory cell infiltration, airway wall cell hyperplasia, and alveolar thickening •↓IL-1β, TNF-α, IL-6 •↑Arg-1 •restore the imbalanced gut microbiota •↑α-diversity and β-diversity •inhibit the phosphorylation of STAT1 in MH-S cells •promote macrophage polarization toward M2 phenotype	(129)
	*Clostridium scindens*	Immune Modulation, Inhibitory metabolites	•enhance epithelial barrier integrity •suppress *S. aureus* enterotoxin production	(130)
	*Deoxycholic acid (DCA)*	Inhibitory metabolites	•↓TNF-α, IL-1β, IL-6, MPO •↓mammary damage, inflammatory parameters •↑ZO-1, Occludin, Claudin-3 •TGR5-cAMP-PKA-NF-κB/NLRP3 pathways	(131)
	Lactomodulin	Antimicrobial peptides	•↓lactomodulin toxicity •↓IL-6, IL-1β, TNF-α	(132)
*K. pneumonia*	*K. oxytoca strain* MK01	Nutritional competition	•They share similar spatial niches and are therefore likely to compete for nutrients. •↓beta-glycosides • CasA enables *K. oxytoca* to outcompete *K. pneumoniae* for beta-glucosides *in vivo*.	(27)
	F18-mix	Nutritional competition	•↑microbial diversity •Gnt-k-dependent gluconic acid metabolism is involved in regulation. •↓gluconate	(19)
	Normal microbiota	Inhibitory metabolites	•↓PH, intracellular acidification •↑acetate, propionate and butyrate	(77)
	high-fiber (HF) dietary carbohydrates	Nutritional competition	•↑microbiome diversity •↑*Lactobacillus johnsonii, Bifidobacterium pseudolongum* and *Lachnoclostridium* •↓Inflammation score	(133)
	Secondary bile acids	Inhibitory metabolites	•Binge-on-chronic alcohol consumption altered the intestinal microbiota •Binge-on-chronic alcohol consumption altered the fecal metabolic profile •Secondary bile acids inhibited *K. pneumoniae* growth •Lithocholic acid inhibited the adhesion of *K. pneumoniae* to Caco-2 cells	(134)
	Taurine	Inhibitory metabolites	•remodel the microbiota •↑α-diversity, β-diversity •↑ *Deltaproteobacteria* •↑bile acid-directed activity •Taurine-derived sulfide inhibits pathogen respiration	(135)
	*Lactobacillus*	Niche exclusion	•↓PH •Lactulose/isomalto-oligosaccharide/inulin and fructo-oligosaccharide can enhance the inhibitory effect of *Lactobacillus* strains against KPC001	(136)
*A. baumannii*	*Bifidobacterium* breve strain Yakult (BbY)	Mucosal barrier	•↓weight loss and mortality •↑acetic acid •↓PH •↓endotoxin levels •↑claudin-1, occludin, and ZO-1	(137)
*P. aeruginosa*	Normal microbiota	Immune Modulation	•remodel the microbiota •improved metabolism and function •↓TNF-α, IFN-γ, IL-6, IL-2, IL-17 •↑Foxp3, IL-10, TGFβ1 •restore Treg/Th17 cell balance	(138)
	Marine prebiotic fucoidans	CDI	•two-partner secretion (TPS) family proteins (TpsA1/CdiA2 and TpsA2/CdiA1) •↑Bacteroides, Enterobacteriaceae and Enterococcaceae	(139)
	*L. plantarum* spp	CDI	•reduce biofilm formation by pathogenic bacteria •disrupt preformed biofilms	(140)
	Normal microbiota	Inhibitory metabolites	•↓Disease score, lung injury •↑Percent survival, cDC1, cDC2 •↑*Muribaculaceae*, α-diversity, β-diversity •↑SCFAs (acetate, butyrate and propionate)	(95)
*C. difficile*	*B.thetaiotaomicron*, *B.vulgatus, P.copri*	Inhibitory metabolites-succinate	•SUCNR1 activation by succinic acid triggers cluster cell proliferation and IL-25 release. •↑IL-25, IL-13 •Type 2 immune response activation enhances the repair and barrier function of the intestinal epithelium	(9)
	*Akkermansia muciniphila* Muc, *Ruthenibacterium lactatiformans* 585-1, *Alistipes timonensis* JC136, *Muribaculum intestinale* YL27, and *Bacteroides* sp. FP24	Nutritional competition	•↓mucosal sugar (NeuAc and GlcNAc) •↑Acetic Acid, Propanoic Acid •↓PH	(21)
	*C. difficile* strain 630	Niche exclusion	•Non-pathogenic *C. difficile* strains inhibit the germination of pathogenic *C. difficile* spores by competing with the cogermination factor glycine rather than with nutritional competition •↓glycine	(25)
	*Bifidobacterium pseudocatenulatum* INIA P815, *Enterococcus faecium* FUA027, and *Streptococcus thermophilus* FUA329	Antimicrobial peptides	•UroA •↓IL-1β, IL-6, TNF-α •↑ZO-1, Occludin, Claudin-4	(105)
	*Phascolarctobacterium*	Nutritional competition, Immune Modulation	•↓succinate •↑IL-22 •↑host N-glycosylation	(141)
	Butyrate	Inhibitory metabolites-succinate	•Inulin, maltodextrin, and xanthan gum are purified MACs that consistently suppress CDI •*C. difficile* fitness is most consistently impacted by butyrate, rather than the other two prominent SCFAs (acetate and propionate)	(142)
	Key butyrate-producing Firmicutes bacteria	Inhibitory metabolites	•Depletion of Firmicutes butyricogenes reduced gut microbiota diversity	(143)
	Ursodeoxycholic Acid (UDCA)	Inhibitory metabolites	•↓Spore germination and outgrowth, growth, and toxin activity •Fecal bile acid metabolome changed significantly •Minimal change to the microbiota •↓IL-1 R1, TLR •FXR/FGF15 pathway	(144)
	*Bifidobacteria*	Niche exclusion	•↓TcdA, TcdB •↓PH •↓IL-6, IL-17, IFN-γ, TNF-α •↑IL-10	(145)
	*Clostridium butyricum*	Inhibitory metabolites, Immune Modulation, Mucosal barrier	•↓succinate •↓TNF-α •↑IL-17A, CD4+ • enhance the gut epithelial barrier function	(146)
*Enterobacter* spp.	*Lactiplantibacillus plantarum* NWAFU-BIO-BS29	Antimicrobial peptides, Mucosal barrier	•↑Plantaricin Bio-LP1 •↓TNF-α, IL-6 and IL-β •TLR4 signaling-pathway •Increased the relative abundance of beneficial-intestinal-bacteria •Improve the intestinal mucosal barrier •↑SCFAs	(74)
	Normal microbiota	Inhibitory metabolites	•↑acetate, propionate, butyrate •↓PH, intracellular acidification •↓use of O2 or NO3	(77)
	Normal microbiota	Inhibitory metabolites, Immune Modulation	•↑l-tryptophan, butyrate, TMA, 3,4-TMAB, 4-TMAP, UDCA, GCA and benzoate • Microbiome metabolites as cytotoxic stressors, inducers of apoptosis and inhibitors of mitochondrial function	(82)
	Butyrate-producing bacteria	Inhibitory metabolites	•↑weight, prolonged survival •↑SCFAs (acetate, butyrate and propionate) •Delayed colonization in SPF-R mice is most prominent in the cecum •↑α-diversity •SCFA producing bacteria within the Firmicutes phylum were significantly elevated	(83)
	*Butyricicoccus, Faecalibacterium, Ruminococcus, Collinsella*, and *Coriobacterium*	Inhibitory metabolites	•↑UDCA •↓TGR5-NF-κB axis •↑SCFAs (acetate) •improve intestinal homeostasis •↓IL-6, TNF-α, IL-1β •↑IL-10 •↑tight-junction-related protein, occludin	(89)
	A mixture of formate, acetate, propionate, butyrate, valerate, isobutyrate, isovalerate, lactate, 5-aminovalerate and ethanol	Inhibitory metabolites	•↓PH •Antibiotic-reduced metabolites (acetate, propionate, butyrate, valerate) inhibit carbapenem-resistant *E. coli*	(147)
	Supernatants of *B. thetaiotaomicron* and *B. adolescentis*	Inhibitory metabolites	•↑SCFAs (acetate, butyrate and propionate) •All bacterial strains were maximally inhibited at pH 5.75	(148)

3,4-TMAB, 3-methyl-4-(trimethylammonio)butanoate; 4-TMAP, 4-(trimethylammonio)pentanoate; Arg-1, arginase-1; cAMP, cyclic adenosine monophosphate; CD, cluster of differentiation; cDC, conventional dendritic cells; CDI, contact-dependent inhibition; FGF15, fibroblast growth factor 15; Foxp3, forkhead box protein P3; FXR, farnesoid X receptor; GCA, glycocholic acid; GlcNAc, N-acetylglucosamine; Gnt-k, gluconate kinase; IFN, interferon; IL, interleukin; MACs, microbiota-accessible carbohydrates; MPO, myeloperoxidase; NaB, sodium butyrate; NeuAc, N-Acetylneuraminic Acid; NF-κB, nuclear factor kappa-B; NLRP3, NOD-like receptor thermal protein domain associated protein 3; PH, potential of hydrogen; PKA, protein kinase A; Reg, regenerating family member; SCFAs, short-chain fatty acid; SPF, specific pathogen-free; STAT1, signal transducer and activator of transcription 1; SUCNR1, succinate receptor 1 Curated; Tcd, toxin C. difficile; TGF, transforming growth factor; TGR5, takeda G-protein-coupled receptor 5; Th, T helper cell; TLR, Toll-like receptors; TMA, trimethylamine; TNF, tumor necrosis factor; Treg, regulatory T cell; UDCA, ursodeoxycholic acid; UroA, urolithin A; ZO-1, zonula occludens-1.

↓: downregulation; ↑: upregulation.

### 3.1 E. faecium

*E. faecium*, a Gram-positive facultative anaerobe, is a commensal member of the human gut microbiota, playing a role in digestion and homeostasis (149). However, under antibiotic pressure, it can transform into a pathogenic organism, particularly in immunocompromised or hospitalized individuals (150). Antibiotic-resistant strains, such as vancomycin-resistant *E. faecium* (VRE), pose significant clinical challenges, causing infections like urinary tract infections (UTIs), bacteremia, and endocarditis. The acquisition of resistance genes through horizontal gene transfer exacerbates treatment difficulties, leading to increased morbidity, prolonged hospital stays, and higher healthcare costs.

Studies have demonstrated that reduced expression of antimicrobial peptides RegIIIβ and RegIIIγ enhances susceptibility to VRE infections (151). Antibiotic-induced disruption of the gut microbiota diminishes RegIIIγ production, impairing VRE control. However, stimulation with the TLR7 ligand riquimod (R848) has been shown to restore RegIIIγ levels, facilitating VRE clearance in antibiotic-treated mice (152). Additionally, Kim et al. identified a four-strain *Blautia producta* consortium that restores resistance to VRE post-antibiotic treatment by producing a lantibiotic similar to nisin-A, which inhibits VRE growth (125). High lantibiotic gene abundance in at-risk patients correlates with reduced *E. faecium* levels, and lantibiotic-producing strains prevent VRE colonization in germ-free mice, highlighting their potential as probiotics. Further research has revealed that *L. murinus* Y74 and *L. plantarum* HT121 reduce VRE colonization and restore microbiota diversity in infected mice (126). *Barnesiella* spp. have also been effective in eliminating VRE colonization and improving survival by reshaping the gut microbiota (153). Butyrate-producing bacteria contribute to microbiota restoration and VRE suppression (127). FMT has emerged as a promising intervention, with studies reporting successful VRE decolonization in numerous patients (154–156). Moreover, *Enterococcus* species can exacerbate *C. difficile* pathogenicity by altering gut metabolism and supporting its growth through amino acids such as leucine and ornithine (157). Thus, modulating the gut microbiota and its metabolites presents a promising strategy for preventing and treating *E. faecium* infections.

### 3.2 S. aureus

*S. aureus* remains a leading cause of severe, life-threatening infections (158, 159), with methicillin-resistant *S. aureus* (MRSA) posing a particularly significant threat. MRSA is associated with high morbidity and mortality rates, especially among hospitalized adults (2, 160). MRSA frequently colonizes the gut, particularly in patients exposed to antibiotics or with critical illnesses, disrupting the normal gut microbiota. Its virulence factors—including enzymes, toxins, and biofilm formation—facilitate tissue invasion, induce inflammation, and impair immune responses, exacerbating infection severity and complicating treatment (161).

MRSA’s ecological adaptations and nutritional competitiveness enhance its ability to colonize the gut and initiate infections. Genetic mutations and structural changes enable MRSA to efficiently metabolize carbohydrates, synthesize its cell wall, and produce energy under low-oxygen conditions, allowing it to thrive in the gut environment. However, the presence of beneficial bacteria can counteract its growth (162). Probiotics have demonstrated potential in reducing *S. aureus* enterotoxin production by altering the gut environment without significantly affecting bacterial growth. For instance, *S. lugdunensis* produces lugdunin, which directly inhibits *S. aureus* growth (128, 163). In MRSA-infected mice, a decline in butyrate-producing bacteria correlates with reduced butyrate levels in the gut and bloodstream. Butyrate supplementation has been shown to restore gut mucosal integrity and enhance immune function, offering a promising therapeutic strategy (129). Additionally, *Clostridium scindens* converts primary bile acids (BAs) to secondary BAs, such as DCA, which may enhance the antimicrobial properties of cell membranes (164, 165). Co-culturing *C. scindens* with colonic cells improves cell viability and strengthens the gut barrier, mitigating damage from *S. aureus* infections (130). DCA has also been shown to reduce *S. aureus*-induced mastitis in mice by activating the TGR5 receptor, which suppresses inflammatory pathways like NF-κB and NLRP3 (131). Dysbiosis impairs DCA production and TGR5 activation, worsening MRSA infections. However, restoring the microbiota with beneficial bacteria, such as *C. scindens* or segmented filamentous bacteria, improves infection outcomes (166). Furthermore, targeting the microbiota through genetic modulation, such as CYP1A1 knockdown, reduces harmful metabolites like cadaverine, offering protection against MRSA-induced sepsis (167). This evidence highlights the crucial role of the gut microbiota in both preventing and treating MRSA infections, offering new therapeutic possibilities beyond antibiotics.

### 3.3 K. pneumonia

*K. pneumoniae* is a highly virulent, antibiotic-resistant Gram-negative bacterium that causes severe infections, particularly in immunocompromised individuals (168). Its pathogenicity is driven by several factors: outer membrane proteins that facilitate adhesion and immune evasion, lipopolysaccharides that induce inflammation and septic shock, and polysaccharides that prevent engulfment by immune cells. These mechanisms complicate infection management and contribute to its persistence. The *K. oxytoca* species complex, a component of the human microbiome, produces enterotoxins such as tilimycin and tilivalline and plays a role in antibiotic resistance (169). Studies by Osbelt et al. have shown that certain *K. oxytoca* strains can reduce gut colonization by MDR *K. pneumoniae* in antibiotic-treated and gnotobiotic mouse models. This effect is largely attributed to competition for carbohydrates, such as beta-glucosides (133), which are critical for promoting resistance. These findings suggest that *K. oxytoca* strains may serve as next-generation probiotics to decolonize *K. pneumoniae* and protect against infections (27). Further research by Shen et al. demonstrated that high concentrations of LCA inhibit *K. pneumoniae* growth and reduce its adhesion to Caco-2 cells (134). Sorbara et al. highlighted the role of the gut microbiota in suppressing MDR *K. pneumoniae* by acidifying the proximal colon, which triggers SCFA-mediated intracellular acidification (77). This process activates PPAR-γ in host epithelial cells, reducing oxygen and nitrate availability, impairing the pathogen’s respiration, and stabilizing HIF-1, which promotes antimicrobial peptide synthesis (110, 170). A recent study revealed that the gut microbiota exposed to *K. pneumoniae* produces sulfide via the taurine pathway, decreasing host cell respiration and preventing pathogen invasion (135). These findings highlight the crucial role of the microbiota in combating *K. pneumoniae* and point to potential therapeutic strategies.

### 3.4 A. baumannii

*A. baumannii* is a formidable opportunistic pathogen, particularly in hospital settings, where its ability to acquire multidrug resistance (MDR) poses a significant threat. It is a leading cause of infections in critically ill patients, including urinary tract infections, bloodstream infections, and ventilator-associated pneumonia, contributing to increased morbidity and mortality (171). While community-acquired infections are less frequently MDR, they can still result in severe outcomes (172).

*A. baumannii* typically colonizes the upper respiratory tract and skin, harboring a wide array of genes that confer MDR, such as those encoding carbapenemases and broad-spectrum β-lactamases. Carbapenem-resistant *A. baumannii* (CRAB) is particularly problematic in intensive care units (ICUs), where it is strongly associated with ventilator-associated pneumonia (171). The CRAB genome is equipped with numerous resistance genes and virulence factors, including efflux pumps, iron acquisition systems, secretion systems, phospholipases, and polysaccharides, which enhance its survival and colonization capabilities (173). CRAB infections are linked to prolonged ICU stays, elevated healthcare costs, and increased antibiotic use (174). The limited treatment options for widespread MDR strains prompted the World Health Organization (WHO) to classify *A. baumannii* as a “Priority Pathogen” in 2018, emphasizing the urgent need for novel antibiotics (175). Antibiotic use increases the risk of *A. baumannii* colonization and infection, likely due to the disruption of commensal bacteria that compete for ecological niches (176). Modulating the gut microbiota may offer a promising strategy to restore microbial balance and limit *A. baumannii* infections. For example, Asahara et al. demonstrated that continuous oral administration of *B. breve* strain Yakult (BbY) improved survival rates and inhibited CRAB growth in infected mice. BbY also helped mitigate intestinal environmental disruptions and maintained barrier function (137). Additionally, a positive correlation between CRAB levels and acetic acid production suggests that gut microbiota metabolites play a critical role in preserving intestinal barrier integrity (177). These findings highlight the potential of probiotic interventions to reduce *A. baumannii* infections, improve patient outcomes, and decrease reliance on antibiotics.

### 3.5 P. aeruginosa

*P. aeruginosa* is a Gram-negative opportunistic pathogen responsible for healthcare-associated infections (HAIs), including chronic obstructive pulmonary disease (COPD), cystic fibrosis, cancer, trauma, burns, sepsis, and ventilator-associated pneumonia (178, 179). It is a leading cause of HAIs globally, and the emergence of antimicrobial-resistant *P. aeruginosa* has led the WHO to classify it as a critical priority pathogen (180). This bacterium develops multidrug resistance (MDR) through mechanisms such as altered outer membrane permeability, efflux pump activity, production of antibiotic-inactivating enzymes, and horizontal gene transfer, making infections increasingly difficult to treat (123, 181). Carbapenem antibiotics, such as imipenem and meropenem, are commonly used against *P. aeruginosa*, but the rise of carbapenem-resistant *P. aeruginosa* (CRPA) poses a significant threat (182). CRPA infections are associated with nearly 700,000 deaths annually, with resistance rates in Europe reaching 12.9% (183), underscoring the urgent need for novel therapeutic strategies.

A key factor in *P. aeruginosa* infections is intestinal colonization prior to ICU admission. Gomez-Zorrilla et al. found that pre-admission intestinal colonization by *P. aeruginosa* increases the risk of subsequent infection by nearly 15-fold (184). Disruption of the gut microbiota, which normally provides colonization resistance through antimicrobial compounds, facilitates pathogen invasion. Gut dysbiosis can impair host immune function, disrupt the intestinal barrier, and increase susceptibility to infections. The gut microbiota plays a critical role in immune modulation, with secretory IgA (sIgA) being a key component of mucosal immunity. sIgA, produced at mucosal surfaces such as the intestines and lungs, protects against pathogens like *P. aeruginosa* (185, 186). However, antimicrobial treatment can reduce IgA levels in the lungs, increasing the risk of respiratory infections (187). Research has shown that certain gut bacteria, such as segmented filamentous bacteria, enhance IgA production and protect against *P. aeruginosa* infection (188). Asymptomatic colonization of the gut by carbapenemase-producing Enterobacterales can lead to dysbiosis, exacerbating the severity of *P. aeruginosa* lung infections by reducing immune cells such as alveolar macrophages and conventional dendritic cells, which are critical for fighting respiratory infections (95). Microbial ecosystem treatment (MET-2), aimed at restoring a healthy gut microbiota, has shown promise in reducing *P. aeruginosa* load and promoting the growth of beneficial bacteria (189). Additionally, the gut-lung axis further illustrates the influence of gut microbiota on lung immunity. Th17 cells, a subset of T-helper cells, play a vital role in protecting the lungs from *P. aeruginosa* infections (190). Wen et al. demonstrated that transplanting host intestinal commensal bacteria restores the balance of Tregs and Th17 cells, enhancing their metabolic functions and providing protection against *P. aeruginosa* pneumonia (138). Thus, restoring gut eubiosis offers promising strategies for treating *P. aeruginosa* infections, potentially improving patient outcomes.

### 3.6 C. difficile

*C. difficile* is a spore-forming, toxin-producing anaerobic bacterium and a leading cause of hospital-acquired diarrhea and pseudomembranous colitis (191). It poses a significant healthcare burden, with over 500,000 infections and approximately 30,000 deaths annually, costing over $1.5 billion each year (192). The primary risk factor is antibiotic use, which disrupts the gut microbiota, promoting *C. difficile* spore germination and increasing infection risk (193, 194). While most patients respond to initial antibiotic treatments, up to 25% experience recurrence, with over 60% of relapsed patients suffering further episodes (195). This recurrent nature complicates management, as antibiotics further disrupt the microbiota, increasing relapse risk.

The gut microbiota plays a central role in regulating *C. difficile* infections through ecological competition and nutrient availability. Non-toxigenic *C. difficile* strains can inhibit toxigenic strains by depleting essential metabolites like glycine (25). Similarly, *Caulobacter* spp. inhibit *C. difficile* growth by reducing luminal succinate, a key metabolite for its proliferation (141). SCFAs, particularly butyrate, particularly butyrate, are critical in protecting against *C. difficile* infection. Butyrate strengthens the intestinal mucosal barrier and reduces inflammation (142, 196). A deficiency in butyrate has been linked to poor outcomes in FMT for recurrent infections (143). BAs, regulated by the gut microbiota, also influence *C. difficile* spore germination and resistance. Farnesoid X receptor (FXR) agonists like obeticholic acid reduce infection severity, while ursodeoxycholic acid (UCA) enhances immune responses against *C. difficile* (144, 197). These findings highlight the potential of BA modulation in managing infections. Emerging therapies targeting the microbiota show promise. Bacteriocins from *Bacillus thuringiensis* and AMPs from *Lactobacillus* species inhibit *C. difficile* growth and spore germination (198, 199). Additionally, *Enterococcus* species, often abundant in *C. difficile* patients, worsen outcomes by promoting toxin production through inflammatory effects (157). These findings suggest that modulation of the microbiota, either through direct interventions such as probiotics or through indirect approaches like BAs manipulation, could offer promising new strategies for preventing and treating *C. difficile* infections.

### 3.7 *Enterobacter* spp.

The genus *Enterobacter*, comprising facultative anaerobic Gram-negative bacilli within the *Enterobacteriaceae* family (200), includes the *E. cloacae* complex (ECC), a significant cause of nosocomial infections, particularly in healthcare settings (123). Carbapenem-resistant *Enterobacterales* (CRE) are especially concerning due to their resistance to broad-spectrum antibiotics, including carbapenems. Risk factors for CRE infections include prior CRE colonization, broad-spectrum antibiotic use, ICU admission, mechanical ventilation, prolonged hospital stays, and indwelling catheters (201). CRE infections are associated with higher in-hospital mortality and longer hospital stays compared to carbapenem-susceptible strains, highlighting the urgent need for effective interventions.

The gut microbiota plays a pivotal role in the development and persistence of *Enterobacter* infections. Studies show that CRE-positive individuals exhibit distinct microbiota profiles, characterized by reduced diversity and increased dominance of *Enterococcus*, *Sphingomonas*, and *Staphylococcus* (202). CRE carriers also show elevated Proteobacteria and reduced Bacteroidetes levels compared to non-carriers (203). These shifts may facilitate CRE establishment and persistence in the gut. Broad-spectrum antibiotics, commonly used to treat infections, disrupt CR, enabling CRE expansion. Antibiotics deplete microbial metabolites that suppress CRE growth while enriching nutrients that support CRE proliferation, perpetuating dysbiosis and infection spread (147). Promisingly, certain probiotics and metabolites have shown potential in mitigating CRE colonization. Supernatants from *C. butyricum*, *E. faecium*, and *L. plantarum* suppress CRE growth in a dose-dependent manner, while those from *B. fragilis* and *B. longum* are less effective (204). Furthermore, SCFAs also differentially inhibit *Enterobacteriaceae* species, suggesting microbial metabolites play a key role in modulating CRE growth (148). These findings underscore the critical role of the gut microbiota in combating *Enterobacter* infections. The potential for microbiota-based therapies, such as probiotics or the restoration of microbial diversity, open an exciting avenue for future research. As our understanding of the gut’s role in resisting *Enterobacter* spp. and other pathogens deepens, such therapies could play a key role in reducing the burden of CRE infections and improving patient outcomes.

## 4 Gut microbiota-based therapies for MDR infections

**Figure 3:**
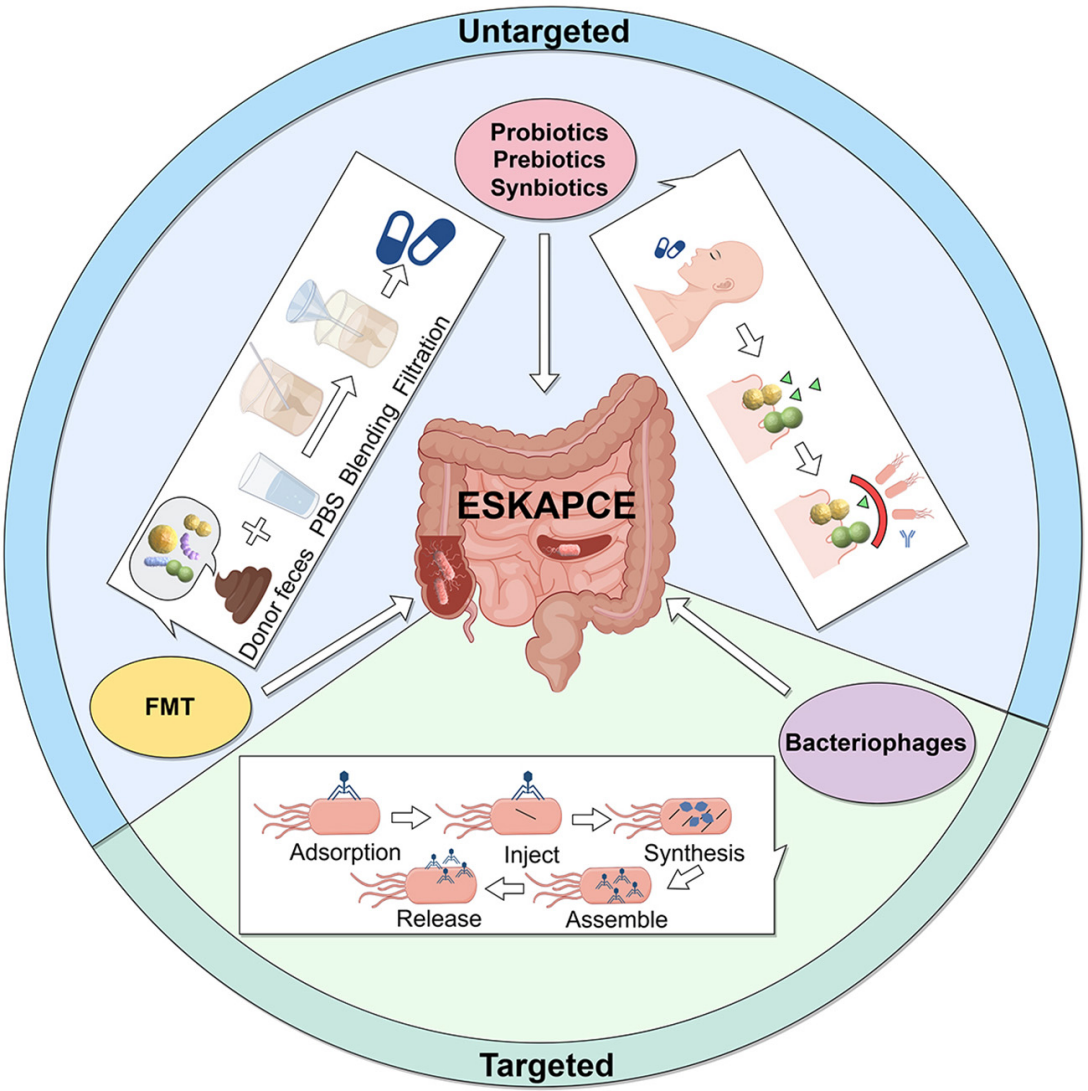
Gut microbiota-based therapies for MDR infections. Probiotics and Prebiotics: Administered in appropriate doses, they prevent pathogen colonization by competing for resources and binding sites, maintaining gut equilibrium, and enhancing immune responses. Fecal Microbiota Transplantation (FMT): Feces from healthy donors is processed and transplanted into patients to restore a balanced gut microbiota. Bacteriophages: Phages kill bacteria through a five-stage process: adsorption, injection, synthesis, assembly, and release, offering a targeted approach to treat bacterial infections.

Advances in microbiome research, powered by omics technologies such as genomics, metagenomics, and metabolomics, have significantly enhanced our understanding of host-microbiota interactions (205). These insights highlight the potential of modulating the gut microbiota to combat MDR infections. By altering the composition and activity of the microbiota, it is possible to reduce the colonization and spread of drug-resistant pathogens, restore microbial balance, and improve gut health. Emerging strategies, including probiotics, prebiotics, synbiotics, postbiotics, FMT, and bacteriophages, are gaining traction as promising tools to address MDR infections (206–208) (**Figure 3**, **Table 3**).

**Table 3:** Probiotics, prebiotics, synbiotics and postbiotics and their mechanisms of action.

Therapies		Pathogenic bacteria	Action mechanism	Major findings	References
Probiotics	*E.coli* Nissle 1917	*Enterococcus faecalis* ATCC 19433	Antimicrobial peptides	• ↑ Bacteroidetocin A (#22), Actifencin (#13) • ↓*Bacteroides*, *Lactobacillus* • ↑Microbial diversity	(69)
	*E.coli* Nissle 1917	*Enterobacteriaceae*	Antimicrobial peptides	• ↑EcN’s microcins (mcmA, mchB) • The absence of mcmA, mchB resulted in significant changes in the intestinal microbial community structure	(70)
	*Ligilactobacillus salivarius* 7247	*Enteritidis* (SE)*,Typhimurium* (ST)	Antimicrobial peptides	• ↑lactic acid • Lactic acid produced by the LS7247 strain increases the permeability of *Salmonella* strains’ outer membrane. • ↑Enterolysin A, Metalloendopeptidase • ↑lantibiotic nisin S, bacteriocin (class IIb) • ATP Leakage	(73)
	*Akkermansia muciniphila*	*Citrobacter rodentium*	Mucosal barrier	• *A. muciniphila* confers infection resistance under fiber-rich conditions and in the absence of other mucin degraders • *A. muciniphila* prevents pathogen invasion by renewing the mucous layer and enhancing tight junction protein expression	(102)
	*Bifidobacterial*	*C. difficile*	Niche exclusion	• ↓TcdA,TcdB • ↓PH • ↓IL-6, IL-17, IFN-γ, TNF-α • ↑IL-10	(145)
	*Clostridium butyricum*	*C. difficile*	Inhibitory metabolites, Immune Modulation, Mucosal barrier	• ↓succinate • ↓TNF-α • ↑IL-17A, CD4+ • enhanced the gut epithelial barrier function	(146)
	*L. plantarum* spp	MDR *Pseudomonas aeruginosa, Staphylococcus aureus* and *Escherichia coli*	Contact-dependent inhibition(CDI)	• reduce biofilm formation by pathogenic bacteria • disrupt preformed biofilms	(140)
Prebiotics	Marine prebiotic fucoidans	*Pseudomonas aeruginosa*	Contact-dependent inhibition (CDI)	• two-partner secretion (TPS) family proteins (TpsA1/CdiA2 and TpsA2/CdiA1) • ↑Bacteroides, Enterobacteriaceae and Enterococcaceae	(139)
	Chitooligosaccharides	Pathogenic *Klebsiella*	Inhibitory metabolites	• ↑acetic acid • ↓propionic, butyric acids • ↓total bacterial population • It did not affect diversity and richness of the gut microbiota. • ↑Bacteroidetes • ↓Proteobacteria, Actinobacteria, Firmicutes/Bacteroidetes	(209)
	Green banana flour	/	/	• ↑Coriobacteriaceae_UCG-002, Turicibacter, Parasutterella, Gastranaerophilales_ge, and RF39_ge • amino acid biosynthesis and secondary metabolite biosynthesis	(210)
	Galacto-Oligosaccharide	*E. coli* O157	Mucosal barrier	• ↓IL-6, IL-1β, TNF-α • ↑MUC2, ZO1, Claudin and Occludin • ↑SCFAs • ↑Microbial diversity	(211)
Synbiotics	*Bifidobacterium breve* strain Yakult (BbY), galactooligosaccharides (GOS)	Multidrug-Resistant *Acinetobacter baumannii*	Mucosal barrier	• ↓weight loss and mortality • ↑acetic acid • ↓PH • ↓endotoxin levels • ↑claudin-1, occludin, and ZO-1	(137)
	*Lactobacillus* with lactulose/isomalto-oligosaccharide/inulin and fructo-oligosaccharide	*K. pneumoniae*	Niche exclusion	• ↓PH • Lactulose/isomalto-oligosaccharide/inulin and fructo-oligosaccharide can enhance the inhibitory effect of *Lactobacillus* strains against KPC001	(136)
Postbiotics	Lactomodulin	MRSA,VRE	Antimicrobial peptides	• lactomodulin toxicity is minimal • ↓IL-6, IL-1β, TNF-α	(132)
	Butyrate	*S. aureus*	Inhibitory metabolites	• ↓lungs displayed inflammatory cell infiltration, airway wall cell hyperplasia, and alveolar thickening • ↓IL-1β, TNF-α, IL-6 • ↑Arg-1 • restored the imbalanced gut microbiota • ↑α-diversity and β-diversity • inhibit the phosphorylation of STAT1 in MH-S cells • promote macrophage polarization toward M2 phenotype	(129)
	D*eoxycholic acid* (DCA)	*S. aureus*	Inhibitory metabolites	• ↓TNF-α, IL-1β, IL-6, MPO • ↓mammary damage, inflammatory parameters • ↑ZO-1, Occludin, Claudin-3 • TGR5-cAMP-PKA-NF-κB/NLRP3 pathways	(131)
	Lactomodulin	*S. aureus*	Antimicrobial peptides	• lactomodulin toxicity is minimal • ↓IL-6, IL-1β, TNF-α	(132)
	Secondary bile acids	*K. pneumonia*	Inhibitory metabolites	• Binge-on-chronic alcohol consumption altered the intestinal microbiota. • Binge-on-chronic alcohol consumption altered the fecal metabolic profile. • Secondary bile acids inhibited *K. pneumoniae* growth • Lithocholic acid inhibited the adhesion of *K. pneumoniae* to Caco-2 cells	(134)
	Taurine	*K. pneumonia*	Inhibitory metabolites	• remodel the microbiota • ↑alpha diversity, beta diversity • ↑Deltaproteobacteria • ↑bile acid-directed activity • Taurine-derived sulfide inhibits pathogen respiration.	(135)

Arg-1, arginase-1; ATP, Adenosine triphosphate; cAMP, Cyclic adenosine monophosphate; CD, Cluster of Differentiation; EcN, Escherichia coli Nissle; IFN, interferon; IL, interleukin; mcm, microcin M; MPO, Myeloperoxidase; MUC, Mucin; NF-κB, nuclear factor kappa-B; NLRP3, NOD-like receptor thermal protein domain associated protein 3; PH, Potential of hydrogen; PKA, protein kinase A; SCFAs, Short-chain fatty acid; STAT1, signal transducer and activator of transcription 1; Tcd, Toxin; TGR5, Takeda G-protein-coupled receptor 5; TNF, tumor necrosis factor; ZO-1, Zonula occludens-1.

↓: downregulation; ↑: upregulation.

### 4.1 Probiotics

Probiotics are live microorganisms that, when administered in adequate amounts, confer health benefits to the host by restoring or maintaining a healthy gut microbiota (212, 213). Common probiotic strains, such as *Lactobacillus*, *Bifidobacterium*, and *Saccharomyces*, exert their effects through the production of antimicrobial substances (e.g., lactic acid, hydrogen peroxide), nutrient competition, and immune modulation (214). These mechanisms reduce infection risk, making probiotics valuable in both clinical and dietary approaches to health promotion and infection prevention.

*Lactobacilli* species, such as *L. plantarum* and *L. rhamnosus*, exhibit antimicrobial activity against pathogens like critical role in infection prevention, with strains like MRSA (215) and VRE (156, 216). These strains enhance immune responses and promote the production of beneficial SCFAs (217, 218). For example, *L. plantarum* increases butyrate-producing bacteria, reduces proinflammatory cytokines, and strengthens intestinal barriers, lowering infection risks (219). Additionally, *L. rhamnosus* produces lactomodulin, which has bactericidal effects on resistant pathogens like MRSA and VRE (132). Certain *Lactobacillus* strains also alleviate antibiotic-associated diarrhea and *C. difficile* infections (124, 220, 221). *Bifidobacterium* species, particularly *B. longum*, improve intestinal barrier function and regulate immune responses, suppressing pathogen growth. For example, *B. longum* JDM301 inhibits toxigenic *C. difficile* growth (145). Probiotic combinations, such as *S. boulardii* with *Lactobacillus* and *Bifidobacterium*, effectively prevent the colonization of MDR pathogens like extended-spectrum beta-lactamase (ESBL)-producing bacteria (222). Pre-treatment with combinations like LactoLevure^®^ (*L. plantarum*, *L. acidophilus*, *S. boulardii* and *B. lactis*) improves survival in rodent models infected with MDR *P. aeruginosa* (223). *C. butyricum*, a butyrate-producing bacterium, plays a key role in gut health by promoting beneficial bacteria and inhibiting pathogens (143). For example, *C. butyricum* 588 enhances antibacterial efficacy against *C. difficile* through immune modulation and reinforcement of gut mucosal barriers (146). Despite their potential, the effectiveness of probiotics varies depending on factors such as strain, dosage, and individual microbiota composition. Challenges in their broader application include unclear molecular mechanisms, strain-specific effects, antibiotic resistance, and stability issues. Metabiotic components may offer solutions to these limitations, advancing the field of microbiota-based therapies.

### 4.2 Prebiotics, synbiotics and postbiotics

Prebiotics are non-digestible dietary compounds that promote the growth of beneficial microorganisms, enhancing gut health (224). By supporting beneficial bacteria like *Bifidobacterium* and *Lactobacillus*, prebiotics help restore microbial balance and suppress MDR pathogens (209). Common prebiotics include human milk oligosaccharides (HMOs), inulin, fructooligosaccharides (FOS), galactooligosaccharides (GOS), and dietary fibers like β-glucan, pectin, and resistant starch (55). These compounds maintain a diverse microbiota, inhibiting pathogen growth through competitive exclusion (210). For instance, GOS reduces *E. coli* adhesion by 70% *in vitro* (211, 225), while fucoidan, a marine prebiotic, promotes *Bacteroides* growth and eliminates *P. aeruginosa* in mice (139). Additionally, 1,5-anhydro-d-fructose (1,5-AF), derived from starch and glycogen, exhibits antioxidant and antibacterial properties, reduces cytokine production, and boosts *F. prausnitzii* growth and NAD biosynthesis genes (226).

Synbiotics combine prebiotics and probiotics to synergistically enhance the survival and colonization of beneficial microbes, improving gut health (224, 227). This approach enhances the efficacy of both components: probiotics utilize prebiotics as growth substrates, while prebiotics are more effectively utilized by targeted microbes. For instance, pre-cultivating *L. plantarum* with xylitol reduces *C. difficile* spore germination and decreases mortality in infected mice from 44% to 22% (228). Similarly, combining *Lactobacillus* with prebiotics like lactulose or isomalto-oligosaccharides prevents colonization by KPC-2-producing *K. pneumoniae* (136). *In vitro*, synbiotics show superior antibacterial activity against MDR pathogens like *A. baumannii* and *E. faecalis*, outperforming probiotics alone (137, 229).

Postbiotics, derived from nonviable bacteria or their metabolic by-products, include bacterial components (e.g., cell walls, enzymes, SCFAs, vitamins, peptides) and paraprobiotics (e.g., peptidoglycan, surface proteins) (213, 230). These components provide immunological benefits, with bacteriocins and AMPs disrupting bacterial membranes. For example, Nisin, produced by *Lactobacillus*, inhibits cell wall formation and enhances penetration when complexed with nanoparticles (231–233). SCFAs from fiber fermentation and secondary BAs inhibit *C. difficile* growth and boost immune defense (234, 235). Lactic acid bacteria-derived cell-free supernatants also inhibit biofilm formation, offering a potential strategy against MDR pathogens like *P. aeruginosa*, *S. aureus*, and *E. coli* (140).

Together, prebiotics, synbiotics, and postbiotics offer a powerful approach to managing MDR infections and restoring gut health. Prebiotics nourish beneficial microbes, synbiotics combine probiotics and prebiotics for synergistic effects, and postbiotics provide antimicrobial and anti-inflammatory benefits. These strategies collectively suppress harmful bacteria, enhance immune responses, and maintain gut microbial balance.

### 4.3 FMT

FMT is emerging as a promising strategy to combat MDR bacterial infections. It involves transferring gut microbiota from a healthy donor to a recipient to restore a balanced, functional microbial community. This restoration enhances CR to MDR pathogens through mechanisms like competitive inhibition, bacteriocin production, and immune modulation (206, 236). FMT has demonstrated significant success in treating recurrent *C. difficile* infections and severe gut dysbiosis (237, 238), with a meta-analysis reporting a 90% success rate for recurrent *C. difficile* infections (239). The FDA-approved FMT product Rebyota (RBX2660) has shown a 78.9% therapeutic success rate in this patient group (240). The success of FMT in *C. difficile* infections has spurred interest in its application to other MDR pathogens, including VRE, MRSA, and carbapenemase-producing *Enterobacter* spp (241, 242). FMT restores immune functions and enhances CR, improving pathogen clearance. For example, FMT has reversed lethal sepsis by restoring butyrate-producing Bacteroidetes (243) and eradicated VRE in both animal models and human patients (153, 244, 245). It has also successfully cleared NDM-1 *K. pneumoniae* and other CREs (246). Additionally, FMT has treated nosocomial MRSA enteritis (247) and recurrent UTIs caused by ESBL-producing *K. pneumoniae* (248). FMT holds considerable promise for managing MDR infections and restoring gut health, particularly in patients with recurrent infections and gut dysbiosis. By improving the gut microbiota, FMT modulates the immune system, enhances pathogen clearance, and provides systemic benefits. However, challenges remain (249, 250), including identifying optimal donor profiles (251), standardizing stool processing and administration protocols, addressing long-term safety concerns, and understanding the mechanisms behind FMT’s efficacy. Clear regulatory guidelines and ethical donor selection are also essential. Overcoming these challenges will enhance FMT’s effectiveness and safety, solidifying its role as a key therapeutic strategy in modern medicine.

### 4.4 Bacteriophages

Bacteriophages (phages) are viruses that specifically infect and lyse bacterial cells, offering a targeted alternative to traditional antibiotics (252, 253). Their specificity minimizes disruption to beneficial microbiota, as they often target only certain bacterial strains (254). After eradicating their bacterial hosts, phages naturally die off, reducing accumulation and toxicity risks. Unlike antibiotics, phages do not affect human cells, avoiding harm to healthy tissue. Additionally, bacterial resistance to phages develops more slowly than to antibiotics, making phage therapy a promising tool against MDR infections.

Phage therapy has evolved into various forms to combat MDR bacteria. Personalized phage therapy isolates specific phages tailored to the infecting bacterial strain, offering customized treatment for MDR infections (255). Phage cocktails, combining multiple phages targeting the same pathogen, enhance efficacy and reduce resistance risks. Phage-driven antibiotics explore how phages can complement traditional antibiotics by weakening bacterial defenses or disrupting protective biofilms (256). Clinical trials and case studies highlight the potential of phage therapy against MDR infections caused by *S. aureus*, *P. aeruginosa*, and *A. baumannii*. For example, a six-phage combination (including E215, E217, PAK_P1, PYO2, DEV, and PAK_P4) targeting *P. aeruginosa* outperformed single-phage treatments (257, 258), while a four-phage mix achieved over 98% efficacy against *S. aureus* and reduced the minimum inhibitory concentrations (MICs) of antibiotics like vancomycin in MRSA biofilms (259, 260). This synergy between phages and antibiotics effectively eliminates persistent infections while preserving beneficial gut microbiota (261).

Phages can also support beneficial bacteria. For example, *E. faecalis* V583 carries a phage that eliminates competing strains (262). Phages like ΦCD27 reduce *A. baumannii* bacterial load without harming non-pathogenic bacteria (194, 263). Phage P3CHA inhibits *P. aeruginosa* biofilm formation in mice (264, 265), though repeated exposure can lead to biofilm resistance (266). A four-phage combination disrupts *C. difficile* biofilms *in vitro* (259, 267). Recent studies highlight phages targeting *K. pneumoniae*, including capsular mutants, showing enhanced lethality in combination therapies (268). Corbellino et al. successfully eradicated MDR *K. pneumoniae* using a custom lytic phage preparation (269). These findings underscore the potential of phage therapy as an effective tool for treating infections caused by MDR bacteria. However, there are still challenges to overcome. Immune responses and bacterial resistance can limit the effectiveness of phage therapy. The immunogenicity of phage capsid proteins may lead to rapid clearance, and bacterial resistance complicates treatment outcomes. Strategies to optimize phage combinations and delivery methods are crucial, and further research is needed to establish standardized protocols and evaluate the long-term safety of phage therapy. Despite these challenges, phage therapy remains a promising approach in the fight against antibiotic-resistant infections.

## 5 Conclusion

The gut microbiota plays a critical yet complex role in combating MDR bacterial infections, presenting both significant challenges and untapped potential. The resistance of MDR pathogens to multiple antibiotics limits the efficacy of conventional therapies, while the intricate variability of the microbiota complicates the development of targeted treatments. The widespread overuse of antibiotics disrupts the microbiome’s balance, promoting the spread of resistance genes and increasing infection risks. Additionally, our incomplete understanding of microbiota-host immune interactions hinders the creation of effective microbiota-based therapies. Despite these challenges, leveraging the gut microbiota has emerged as a pivotal strategy in addressing MDR infections and the growing threat of AMR. A range of therapeutic approaches—including probiotics, prebiotics, synbiotics, postbiotics, FMT, bacteriophage therapy, and CRISPR-Cas-based engineered strains—show promise in modulating the microbiota to enhance host resistance. These interventions aim to restore microbial balance, strengthen protective functions, and mitigate AMR effects.

However, the complexity of the microbiota and the need for precision medicine necessitate further research into personalized microbiota-based therapies and the development of novel antibacterial agents. Future studies should focus on tailoring interventions to individual microbiome profiles, ensuring the long-term safety of microbiota modulation, and integrating microbiome-based approaches with new antibacterial compounds. Advanced technologies, such as artificial intelligence and big data analytics, can enable more precise and effective treatments for MDR infections. Ultimately, overcoming AMR requires a collaborative, multidisciplinary approach to translate innovative strategies into scalable, safe, and practical solutions. By harnessing the full potential of the gut microbiota, we can pave the way for sustainable and effective approaches to managing resistant infections, improving public health outcomes, and addressing the global antibiotic resistance crisis.
